# The Use of Warm Air for Solvent Evaporation in Adhesive Dentistry: A Meta-Analysis of In Vitro Studies

**DOI:** 10.3390/jfb14050285

**Published:** 2023-05-20

**Authors:** Rim Bourgi, Louis Hardan, Carlos Enrique Cuevas-Suárez, Francesco Scavello, Davide Mancino, Naji Kharouf, Youssef Haikel

**Affiliations:** 1 Department of Restorative Dentistry, School of Dentistry, Saint-Joseph University, Beirut 1107 2180, Lebanon; rim.bourgi@net.usj.edu.lb (R.B.); louis.hardan@usj.edu.lb (L.H.); 2 Department of Biomaterials and Bioengineering, INSERM UMR_S 1121, University of Strasbourg, 67000 Strasbourg, France; mancino@unistra.fr (D.M.); youssef.haikel@unistra.fr (Y.H.); 3 Dental Materials Laboratory, Academic Area of Dentistry, Autonomous University of Hidalgo State, San Agustín Tlaxiaca 42160, Mexico; cecuevas@uaeh.edu.mx; 4 IRCCS Humanitas Research Hospital, 20089 Rozzano, Milan, Italy; 5 Department of Endodontics and Conservative Dentistry, Faculty of Dental Medicine, University of Strasbourg, 67000 Strasbourg, France; 6 Pôle de Médecine et Chirurgie Bucco-Dentaire, Hôpital Civil, Hôpitaux Universitaire de Strasbourg, 67000 Strasbourg, France

**Keywords:** dentine, silane, solvent-based, total-etch, warm air

## Abstract

Any excess solvent from dental adhesive systems must be eliminated prior to material photopolymerization. For this purpose, numerous approaches have been proposed, including the use of a warm air stream. This study aimed to investigate the effect of different temperatures of warm air blowing used for solvent evaporation on the bond strength of resin-based materials to dental and nondental substrates. Two different reviewers screened the literature in diverse electronic databases. In vitro studies recording the effect of warm air blowing to evaporate solvents of adhesive systems on the bond strength of resin-based materials to direct and indirect substrates were included. A total of 6626 articles were retrieved from all databases. From this, 28 articles were included in the qualitative analysis, and 27 remained for the quantitative analysis. The results of the meta-analysis for etch-and-rinse adhesives revealed that the use of warm air for solvent evaporation was statistically significantly higher (*p* = 0.005). For self-etch adhesives and silane-based materials, this effect was observed too (*p* < 0.001). The use of a warm air stream for solvent evaporation enhanced the bonding performance of alcohol-/water-based adhesive systems for dentin. This effect seems to be similar when a silane coupling agent is submitted to a heat treatment before the cementation of a glass-based ceramic.

## 1. Introduction

Adhesive systems contain resin monomers with hydrophilic and hydrophobic characteristics, polymerization modulators, and a high concentration of solvents [1]. Organic solvents act as diluting agents that improve wetting and the infiltration of resin monomers into the dentinal surface [2]. It is crucial that any excess solvent must be eliminated from the dental substrate by means of air drying prior to the photopolymerization of the applied adhesive, in addition to allowing time between these two processes [3]. A presence of residual solvents might affect the polymerization of monomers and hinder the integrity of the bond, creating unwanted pathways for voids inside the adhesive interface and causing a decrease in the bond strength [4,5].

Various approaches favoring solvent evaporation have been proposed. These include an increased adhesive application time [6], multiple adhesive layers [7], delayed light curing [8], vigorous adhesive rubbing [9], longer exposure duration of bonding systems [10], and extended air drying [11]. Additionally, using a warm air stream was found to sufficiently evaporate solvents included in an adhesive system [12]. Authors have experimented on external sources for heating before photopolymerization, ranging within biologically acceptable limits [13,14,15], leading to immediate monomer conversion gains while reducing the concentration of the final solvent in the adhesive system [16].

The application of a warm air stream was found to adequately evaporate solvents from an adhesive system [12]; this might clinically increase the dentin bond strength. Authors have experimented on external sources of heating before photopolymerization, ranging within biologically acceptable limits [12,13,14,15,17,18], resulting in immediate monomer conversion gains while reducing the concentration of the final solvent in the adhesive system [16]. Furthermore, this approach enhances resin infiltration into the decalcified dentin, consequently improving dentinal bond strength. It is important to mention that a warm air stream within the thermal tolerance zone of the dentin pulp organ (29–56 °C) will not yield any undesirable pathological effects on the dentin pulp organ and dentin will respond physiologically to warm air blowing [19,20,21]. A previous meta-analysis proposed that the dentin bond strength of adhesive systems might be improved by using warm air streams for solvent evaporation [22]. Several air temperatures for solvent evaporation, comprising 37 °C, 38 °C, 50 °C, 60 °C, and 80 °C, were recognized in the aforementioned review. Approximately 40 °C and 60 °C warm-air-drying temperatures were considered efficient for improving solvent evaporation. Nonetheless, the 60 °C temperature was more favorable in terms of stable bond strength and reduced bond degradation [22]. Fittingly, a better description of the gold-standard temperature for air drying should be of great attention.

The use of warm air for an adequate evaporation of solvents has been also explored for silane-based products [23]. In a clinical situation, silanes reduce the contact angle, leaving a film with a thickness ranging between approximately 10 and 50 nm [24]. The outcomes of a study support the theory that silane is capable of raising the surface wettability, causing the formation of chemical bridges with substrates covered by hydroxyl groups (OH) (e.g., glass or quartz fibers) [25]. Furthermore, it has been conveyed that the silane coupling agent significantly improves the bond strength between the fiber posts and the composite core build-up materials [26].

Several layers or a thick film of silane lead to internal cohesive tendencies, and eventually bond failure. Due to this, clinically, an ultrathin silane layer is required to enhance bond strength [27]. An approach to improving its implementation includes thermal drying with temperatures ranging between 50 and 100 °C of silanes once applied to substrates. This permits the evaporation of vehicles, thus accelerating the condensation on the surface and promoting the efficient formation of covalent bonds [28]. However, silane heat treatment at high temperatures (70–80 °C) may not be practical for clinical practices, but a warm air stream (38 °C) acceptable to patients can be used to aid in solvent evaporation and the products of the reaction on the silane-treated surface, leading to a dried surface [29].

The purpose of this manuscript was to investigate the effect of different temperatures of warm air blowing used for solvent evaporation on the bond strength of resin-based materials to dental and nondental substrates. Accordingly, the null hypothesis of the present systematic review and meta-analysis was that the use of a different temperature of warm air stream for solvent evaporation does not affect the bond strength of resin-based materials to dental and nondental substrates.

## 2. Materials and Methods

### 2.1. Protocol and Registration

This systematic review was registered in the Open Science Framework under the identifier DOI 10.17605/OSF.IO/JUQT5 and it respected the suggestions of the Preferred Reporting Items for Systematic Reviews and Meta-Analysis (PRISMA) statement.

### 2.2. Information Sources and Search Strategy

The search strategy (Table 1) was firstly described for the MEDLINE database using keywords for each concept of the following PICOT strategy: population, permanent enamel and dentin, and indirect substrates; intervention: application of warm air for solvent evaporation; control, application of room-temperature air; outcome, bond strength; and type of studies, in vitro studies. The MEDLINE search strategy was adapted to other electronic databases, including Scielo, Web of Science, Scopus, and Embase. Additionally, the first 100 results of Google Scholar were also consulted.

### 2.3. Selection Process and Data Collection Process

After running a search strategy, an online software program (Rayyan, Qatar Computing Research Institute, HBKU, Doha, Qatar) was used to store the files from all databases, and for duplicate detection. The same software program was used for evaluating the title and abstract of the articles. This phase was carried out by two independent reviewers to check whether they encountered the following inclusion criteria: (1) in vitro studies recording the effect of the use of different temperatures of warm air blowing to evaporate solvents from an adhesive system on the bond strength of resin-based materials to enamel, dentin, glass-based ceramics, oxide-based ceramics, metal alloys, and indirect composites; (2) evaluated the bond strength of adhesive systems to the aforementioned substrates with 2 antagonists: composite resin or resin-based cement; (3) comprised a control group; (4) included mean and standard deviation (SD) data in MPa on shear, microshear, microtensile, and tensile bond tests; and (5) available in the English, Spanish, or Portuguese language. Case series, case reports, pilot studies, and reviews were omitted.

Each adequate article received a study identification, merging the last name of the first author with the year of publication. The same two referees reviewed and categorized data, such as the material tested, the solvent contained in the material, the temperature of warm air stream used, the substrate tested, and the bond strength test.

### 2.4. Quality Assessment

The methodological quality of, respectively, integrated manuscripts was evaluated independently by two authors (L.H. and R.B.). The risk of bias in individual articles was considered via the description of the following factors: specimens’ randomization, single operator, operator blinded, control group, standardized specimens, failure mode, manufacturer’s instructions, sample size calculation, and coefficient of variation. If the article included the factor, the study received a “Yes” for that specific parameter. In the case of missing data, the parameter received a “No”. The risk of bias was classified regarding the sum of “Yes” answers received: 1 to 3 denoted a high bias, 4 to 6 medium, and 7 to 9 showed a low risk of bias.

### 2.5. Statistical Analysis

A meta-analysis was executed by means of a software program (Review Manager version 5.3.5; The Cochrane Collaboration, Copenhagen, Denmark). A random-effect model was used, and estimates were acquired by comparing the standardized mean difference between the bond strength values for the groups where a warm air stream was used against the control group in which a room-temperature air stream was considered. The bond strength from etch-and-rinse (ER), self-etch (SE), and silane-based materials was analyzed separately. For each analysis, subgroups were formed considering the type of solvent used for the adhesive formulation. A *p*-value < 0.05 was considered statistically significant. The heterogeneity was designed by means of the Cochran Q test and the inconsistency I^2^ test.

## 3. Results

**Figure 1:**
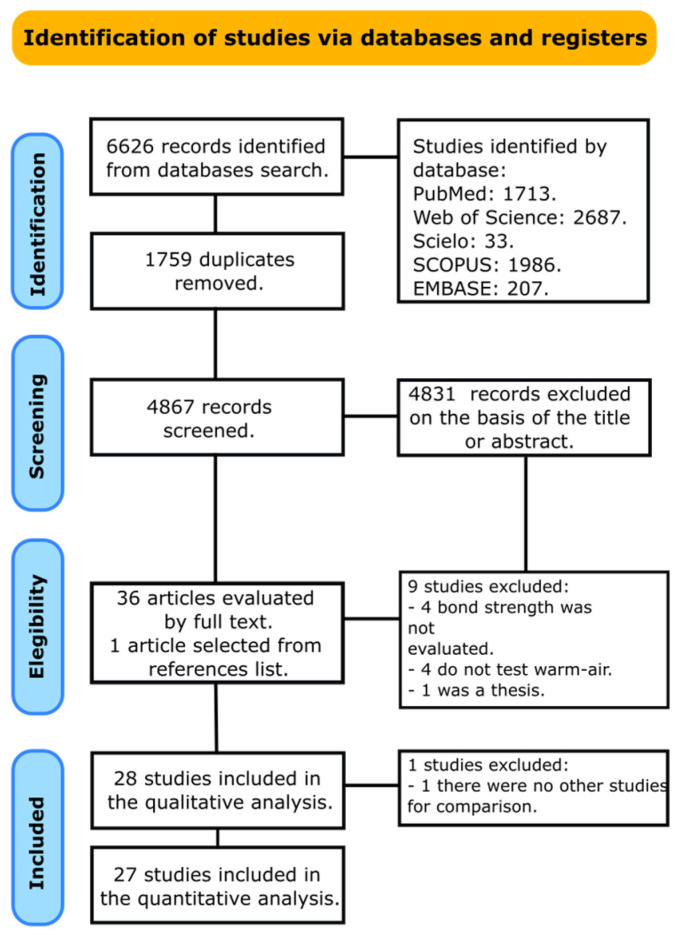
Study selection according to the PRISMA statement.

A total of 6626 articles were retrieved from all databases. Figure 1 shows the flowchart that summarizes the study selection according to the PRISMA statement. A total of 4867 documents were screened by title and abstract after duplicate removal. From these, 4831 were excluded after the initial screening. Thirty-six studies were chosen for full-text reading, and one article was included after hand searching the references from these documents. After reading the full text, nine articles were excluded due to several reasons: in (4), the bond strength was not evaluated [18,30,31,32]; in (4), a warm air stream was not tested [33,34,35,36]; and (1) was a thesis [37]. Then, a total of 28 articles were included in the qualitative analysis [12,13,14,16,20,21,23,24,29,38,39,40,41,42,43,44,45,46,47,48,49,50,51,52,53,54,55,56]. One study was excluded from the meta-analysis because there were no more studies for comparison [53].

The features of the included articles are recapitulated in Table 2 and Table 3. Numerous warm-air temperatures, including 37 °C, 38 °C, 50 °C, 60 °C, and 80 °C, were acknowledged in the present review for solvent evaporation. Most of the included articles assessed the bond strength of both SE and ER adhesive systems. Universal adhesives were found too; however, due their composition, these adhesives were considered as SE adhesives. Different ceramic primers were evaluated too; all of them had a silane coupling agent within its composition. All studies immediately evaluated the bond strength, but only two tested this property after some aging. Microtensile bond strength was the most commonly used test to evaluate bond strength; other tests such as shear and push-out were used too.

**Figure 2:**
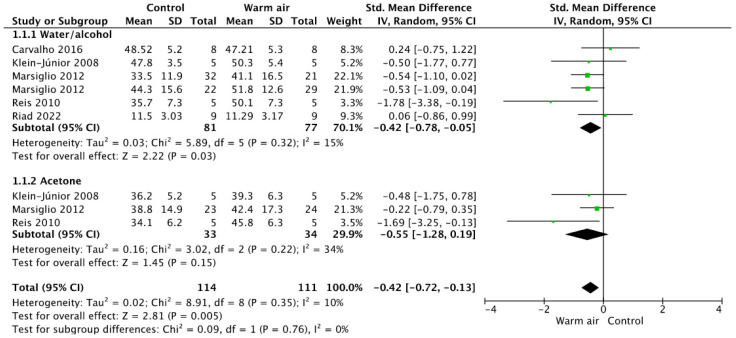
Forest plot showing the effect of warm air stream on the bond strength for etch-and-rinse adhesives.

The outcomes of the meta-analysis for ER adhesives are accessible in Figure 2. The overall analysis revealed that the use of warm air for solvent evaporation was statistically significantly higher (*p* = 0.005). This effect was observed only for the adhesives based in water/alcohol (*p* = 0.03), while for acetone-based adhesive systems, the use of warm air did not increase the bond strength (*p* = 0.15).

**Figure 3:**
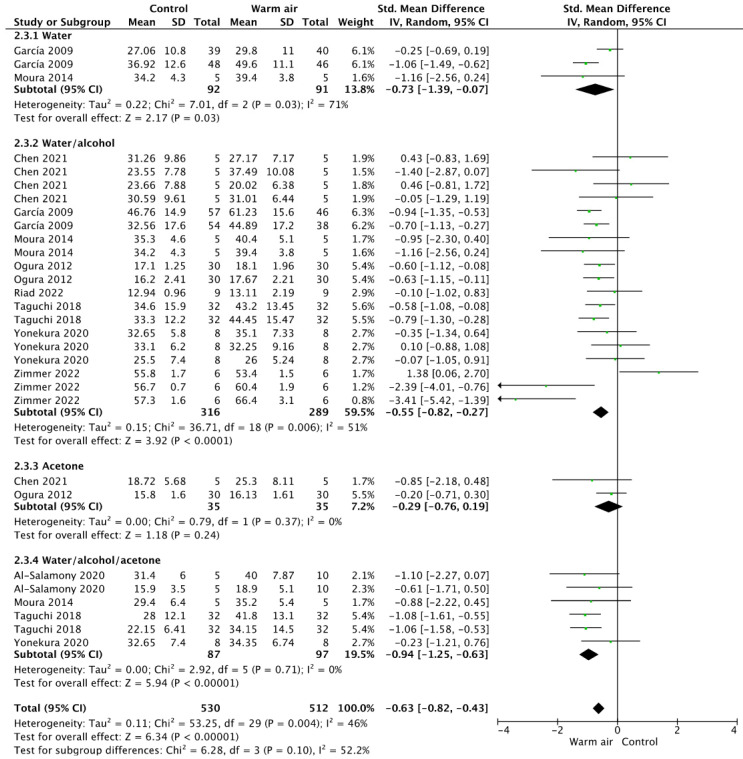
Forest plot showing the effect of warm air stream on the bond strength for self-etch adhesives.

For SE adhesives (Figure 3), the overall analysis privileged the use of warm air (*p* < 0.001). In the subgroup analysis, the use of a warm air stream allowed statistically significantly higher values for the water- and water/alcohol-based adhesive systems (*p* = 0.03, and *p* < 0.0001). This effect was not perceived for the acetone-based products (*p* = 0.24).

**Figure 4:**
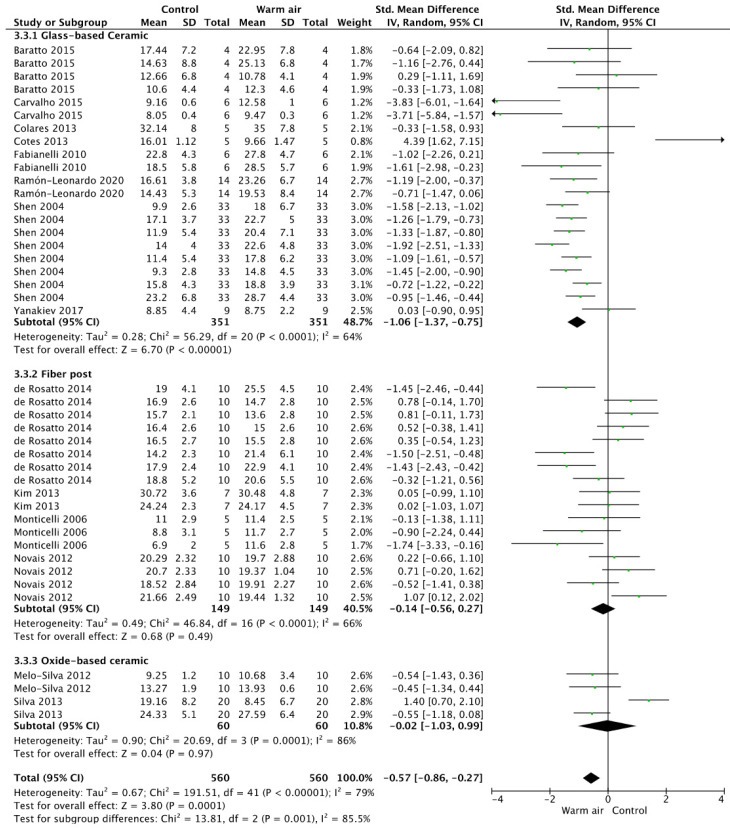
Forest plot showing the effect of warm air stream on the bond strength for silane-based materials.

Finally, in Figure 4 the meta-analysis for the silane-based materials is shown. The overall analysis privileged the use of a warm air stream for solvent evaporation (*p* = 0.001). When analyzing the subgroups, it could be observed that this result was only valid when the silane was applied in glass ceramic materials (*p* < 0.001), while for glass fiber posts and oxide-based ceramics, the use of a warm air stream did not represent any advantage (*p* = 0.49, *p* = 0.97).

Table 4 shows the outcomes from the methodological quality evaluation. Most of the included articles recorded between a medium and low risk of bias. Some of the studies failed to report the specimen randomization, operator blinded, single operator, and sample size calculation factors.

## 4. Discussion

This article aimed to evaluate the effect of different warm-air temperatures used for solvent evaporation on the bond strength of resin-based materials to dental and nondental substrates. For ER or SE adhesives, and for silane as well, the use of a warm air stream for solvent evaporation enhanced the bond strength of direct and indirect restorations. This improvement is directly related to the adhesive system’s base solvent and the tested substrate to which the resin-based materials adhere. The bond strength was enhanced when using a warm air stream for water/alcohol-based systems. On the other hand, the same result was not witnessed for the acetone-based adhesive. For glass-based ceramics, the bond strength was enhanced when a warm air stream was used. In contrast, this enhancement was not observed for fiber post and oxide-based ceramics. Based on the obtained results, the bond strength of the tested resin-based materials improved when using a warm air stream for solvent evaporation. Thus, the null hypothesis tested in this systematic review and meta-analysis was rejected.

Solvent evaporation is a very crucial step in any adhesive system application [13]. That said, solvents and water should be properly eliminated from the dental surface prior to light polymerization. To achieve this evaporation, researchers suggest a process known as air drying for dental adhesion. However, such a task is complicated since the density of the monomer rapidly rises as solvents and water are evaporating [57]. This limits the evaporation capacity and results in reduced water and solvent elimination [58]. However, the exact amount of unevaporated solvents is unspecified; yet, what is known is that this excess can lead to the deterioration of the hybrid layer, as well as a negative impact on the long-term bond performance [59,60]. Other complications include nanoleakage due to the pathways created, in addition to a reduced polymerization of resin monomers [61,62].

The ability of solvents to evaporate is reliant on vapor pressure (mmHg), which is defined as the value of the pressure needed for a liquid to transform into its gaseous state [63,64]. The vapor pressure of dissimilar solvents differs based on the value of the vapor pressure for each solvent, for example, the vapor pressure of acetone was higher (184 mm Hg at 20 °C) than that of water (17.5 mm Hg) and ethanol (43.9 mm Hg) [1,61,65]. It is worth noting that solvents can be bound to the collagen matrix, which reduces evaporation. This explains why ethanol-based bonding systems can retain a bigger number of organic/water mixtures than acetone-based bonding systems following evaporation [66]. Yet, assuming the temperature of air drying was the same for all the solvents, the boiling temperature can still affect the volatility of solvents; in this case, at a lower boiling temperature, ethanol-based adhesive systems will be easier to store than acetone-based adhesive systems [42,67].

Adhesive systems containing a mixture of water and alcohol showed an improvement in the bond strength after a stream of warm air was applied. Hydroxyl group interactions occurring between water and ethanol lead to higher boiling temperatures, which hinder the evaporation of the solvents [61]. A previous study deduced that the percentage of the unevaporated mixture is nearly 13% after a full minute of evaporation [61]. The excess solvents alter the bond strength and the degree of conversion of the adhesive, acting like a plasticizer [68]. Additionally, air drying at a higher temperature increases the kinetic energy of the molecules found inside the adhesive system, which promotes a stronger molecular vibration and alters the bonding process between the polar groups of the resin monomer and the solvent [69]. This in turn enhances solvent evaporation, facilitates polymerization, and strengthens the bond between the adhesive system and the dental substrate [70].

Aside from the air-drying temperature, prolonging the air-drying time also plays an important part in improving the evaporation of the solvents [60,71]. An insufficient air-drying time weakens the bond strength due to the presence of residual solvents such as water and ethanol, which inhibit monomer penetration and polymerization [72,73]. However, the concentration of the solvent does not have a direct impact on the degree of cure of the adhesive [74]. This shows that there should be a threshold level of residual solvents to encourage an improved conversion. Ethanol-based adhesives are a good example of the previous statement. Regarding the amount of solvent, an ideal percentage would be less than 10%. This percentage is not estimated as a remaining solvent after evaporation, but rather incorporated purposefully inside the adhesive bottle depending on the desired amount [47]. The assumption should be that the ideal amount required for the cure is less than that of the remaining solvents after evaporation [75]. All in all, warm air drying decreases the viscosity of the monomers and increases their temperature, which facilitates their diffusion into the dental substrate and leads to a stronger resin bond, justified by the findings of this meta-analysis.

Regarding acetone-based adhesives, warm air stream application did not enhance the bond strength. Acetone is known for its poor hydrogen bonding ability to water and to monomers inside the adhesive bottles [61,76,77]. This is due to its high volatility and vapor pressure [78]. Thus, acetone can be easily eliminated from the adhesives, especially after repeated usage and after storage, which alters its shelf-life [79,80]. Normally, adhesives containing acetone possess a lower monomer/solvent ratio, which dictates multiple layer applications for a better bond integrity [58]. Although, it should be noted that acetone evaporates 5 min after the application, but this is clinically unacceptable. Still, other components can also influence the evaporation time despite the solvent being a crucial factor [21]. Moreover, experimental acetone-based adhesives evaporated at the same rate after a room-temperature or 40 °C air stream was applied [69]. This might prove why the application of warm air did not enhance the bond strength of the acetone-based adhesives in a significant manner.

For the glass-based ceramics, the bond strength was improved when a warm air stream was used. On the contrary, this enhancement was not observed for the fiber post and oxide-based ceramics. The presence of a consistent bond is one of the main aspects of a successful all-ceramic restoration [81]. This requires the integration of all the different parts into one comprehensible and clear system [82]. Etching with hydrofluoric acid (HF) is the preferred process to condition the ceramic restoration surface [83], after which a silane coupling agent is applied to ensure a strong bond [81,84,85,86]. HF dissolves the glassy part of the ceramic network by generating surface pits [87]. The application of silane on the etched ceramic surface enhances the wettability and produces a covalent bond between the luting cement and the ceramic [88]. Both mechanisms can lead to micromechanical attachment as well as chemical bonding [44,89]. Silane can provide a mean for a chemical bond to occur at the silica-based ceramic surface by binding inorganic components to organic ones to achieve “coupling” [90]. Silanol is produced after the inorganic components of silane hydrolyze. This produces a metal hydroxide, also known as a siloxane bond, with the inorganic group. A covalent bond is produced when the organic component of silane causes a reaction with the organic material. Consequently, the organic and the inorganic materials will be bound to each other after heat treatment [91].

Heat treatment can evaporate alcohol and water as well as other products, while aiding with the silane–silica condensation reaction and the covalent bond formation [88,89,92]. This enhancement in the procedure of silane bonding can lead to a reliable and durable bond to the indirect substrate, making micromechanical retention unnecessary [89]. HF can be avoided due to reasons that include its high toxicity [93], its ability to create insoluble silica-fluoride salts which might be retained on the surface and weaken the bond strength [50,94], in addition to the fact that some ceramic structures do not require HF [95]. Despite that, the silane bond should be adequate to justify the absence of HF. This can be performed using heat treatment. After silane application, the restoration can be heat-treated for 2 min at 100 °C in an oven, allowing the removal of solvents along with other by-products on the ceramic surface, thus fulfilling the reaction of condensation between silane and silica. After that, the adhesion will be stronger and more effective due to the formation of many covalent bonds on the surface of the ceramic [46,96]. The same result can be obtained by the application of hot air at 50 ± 5 °C for 15 s [97]. A previous study attempted to determine which silane heat treatment process yields a better bond strength value. The researchers of that study found that even without etching, the heat treatment of silane containing a functional monomer called 10-Methacryloyloxydecyl dihydrogen phosphate (10-MDP) enhanced the bond strength between the ceramic and the luting agent, whether it was heated in an oven (100 °C for 2 min) or with hot air (50 ± 5 °C) [40]. Undergoing heat treatment at a temperature of 100 °C showed that when applying three layers of the silane on the ceramic surface, these multiple layers become consolidated into a monolayer, which increases the bond strength of the composite to the ceramic [88,92]. This heat treatment is also capable of evaporating the solvent and the volatile products formed after the condensation reaction of the silanol groups [88]. Another study carried out by Moniticelli et al. proved that warm air drying at a temperature of 38 °C increased the efficiency of the silane coupling agents when bonding ceramic to composite resin [50]. Additionally, a study concluded that, after treatment through surface roughening, the shear bond strength was significantly improved with heat treatment at a temperature of 100 °C for 60 s [88]. Likewise, Shen et al. discovered that silane drying with a stream of warm air (45 °C) improved the bond strength of glass ceramic [29]. Thus, the bond between the ceramic and the resin is significantly strengthened when a drying step is added to the application of silane at 100 °C [46]. Heat treatment at high temperatures (70–80 °C) for silane coupling agents might not be suitable for dental chair-side operations. Yet, a warm air stream (38 °C) can be tolerated to a better extent by the patient [29]. Warm air streams can aid in solvent evaporation while promoting the condensation reaction of silanol groups. As demonstrated in this meta-analysis, warm air drying may stimulate the formation of a chemical bond, not only in ceramic, but in the silane group as well [29]. Thus, the possession of a mini blow dryer may be viable.

When the tooth is endodontically treated and an essential part of the coronal tooth substance is lost, in addition to being prone to masticatory forces, a post is required inside the root canal to retain the end restoration [98]. Fiberglass has been used in recent years as a type of post since it can improve the performance of the restoration with a better stress distribution on the residual tooth when compared to ceramic or metallic posts [99]. However, a previous study concluded that the most common reason for the clinical failure of this restoration is the detachment of the fiberglass post from the root dentin [100].

A vital step in any clinical situation is to undergo silanization on the post surface after treating it chemically or mechanically [101]. The key factors influencing the efficiency include: the silane type, the pH, the solvent’s content, the molecule of the silane, the molecule dimensions, and the mode of application such as drying settings, duration between the application of the silane and the adhesive resin, and the environmental humidity and temperature [102]. The rule is that the molecules known as organosilanes are bifunctional; one end is able to react with the inorganic part of the fiberglass and the other end can copolymerize with the resin which is organic [102].

The bond strength to fiberglass posts might be influenced by the composition of silane and the temperature used for air drying [88]. The temperature will act as a catalyst for the silane reaction, which accelerates the chemical interaction that occurs between the silane and the inorganic surface. Moreover, solvent evaporation plays a significant role in silane performance by encouraging silane wetting, but, an inadequate solvent evaporation may negatively affect the interaction with fiberglass [103]. Warm air thus aids with the aforementioned process [29]. Multiple opinions exist regarding the use of warm air. One study showed that the application of warm air on silane at a temperature of 38 °C improved the bond strength of the composite resin to the fiber post [50]. However, previously, it was argued that air drying at room temperature (23 °C) is more effective than at a high temperature (60 °C) when bonding resin cement to fiber-reinforced composite posts [48]. The main speculation is that despite the improvement that heating can have on solvent evaporation, the extent to which the evaporation is enhanced is possibly linked to the volatility of the specified solvent, such that the bond strength may be reduced after heating [51]. When the temperature is raised on the fiber post, the reaction may be finalized and the remaining solvent may be evaporated adequately, thus enhancing the bond strength of resin-based materials to indirect restoration, and which does not support the findings of this review [45].

For oxide-based ceramics, the use of warm air did not improve the bond strength of indirect restorations. Few articles have researched this topic [49,54]. The researchers involved in this field experimented on the effect of silane air-drying temperature (room temperature and 70 °C) on the adhesion of oxide-based ceramics (feldspar and yttrium-stabilized zirconia) and found that the increased air-drying temperature enhanced the bond resistance of zirconia ceramics, but not feldspar ceramics [49]. This is justified by the different structures of both materials tested. Different to ceramic, zirconia is comprised of small particles with the absence of a glassy stage which does not retain the resin bonding agents in a satisfactory way [104]. Alternative methods to enhance the adhesion of zirconia include aluminum oxide gushing and silica tribochemical coating [105]. The latest technique is warm air which did not match with the findings of this meta-analysis as no improvement in the bond strength was observed [49].

Finally, it should be highlighted that the use of a warm air stream for solvent evaporation can enhance the bond strength of adhesive systems and silane-based materials; thus, this review might identify that in a clinical situation, this variable should be explored, leading to long-term direct and indirect adhesive restorations. Scientists must be encouraged to design randomized clinical trials in order to explore if the use of warm air for restorative procedures is reliable.

## 5. Conclusions

Within the limitation of this systematic review of in vitro studies, it could be concluded that the use of a warm air stream for solvent evaporation enhanced the bonding performance of alcohol-/water-based adhesive systems when applied to dentin. According to the studies examined, the gold-standard temperature of a warm air stream should be around 50 and 60 °C when applied to dentin. In addition, this improvement seems to be similar when a silane coupling agent is submitted to heat treatment before the cementation of a glass-based ceramic. For indirect substrates, a temperature above 60 °C is recommended for the warm air stream.

## Figures and Tables

**Table 1:** Search strategy used.

Search	Keywords
# 1	Dental Bonding OR Self-Cured Dental Bonding OR Chemical-Curing of Dental Adhesives OR Chemical Curing of Dental Adhesives OR Dentin-Bonding Agents OR dental primer OR Dental Materials OR Dental Material OR dental resin OR Dental Resins OR bonding interface OR adhesive OR Dentin-Bonding Agents OR Dentin Bonding Agents
# 2	warm air OR temperature air OR air-blowing OR air-drying OR air-stream
# 3	# 1 and # 2

**Table 2:** Demographic and study design data of the included studies regarding direct substrates.

Study	Year	Material Tested and Category of the Material	Solvent Contained within the Material	Temperature of Warm Air Stream	Substrate Tested	Bond Strength Test
Al-Salamony [38]	2020	Optibond XTR (Kerr Co., Orange, CA, USA): Two step self-etch adhesive	Water, ethanol, and acetone	39 and 50 °C	Human dentin (Permanent)	Microtensile bond strength (μTBS)
		Optibond All-In-One (Kerr Co., Orange, CA, USA): One step self-etch adhesive	Water, ethanol, and acetone			
Carvalho [41]	2016	Adper Single Bond 2 (3M ESPE, St. Paul, MN, USA): Two-step total-etch adhesive system	Ethanol, water	50 °C	Human dentin (Permanent)	μTBS
Chen [42]	2021	Adhese Universal Vivapen (Ivoclar Vivadent, Schaan, Liechtenstein)	Ethanol, water	60 ± 2 °C	Human dentin (Permanent)	μTBS
		Gluma Bond Universal (Heraeus Kulzer, Hanau, Germany)	Acetone, water			
		All Bond Universal^®^ (Bisco, Schaumburg, USA)	Ethanol, water			
		Single Bond Universal (3M ESPE, St. Paul, MN, USA)	Ethanol, water			
		Clearfil Universal Bond (Kuraray Noritake Dental Inc., Osaka, Japan)	Ethanol, water			
Garcia [47]	2009	Clearfil SE Bond (Kuraray Co. Inc., Osaka, Japan): Two-step self-etch	Ethanol, water	38 °C	Human dentin (Permanent)	μTBS
		Clearfil SE Protect (Kuraray Noritake Dental Inc.). Two-step self-etch	Water			
		Adper Prompt-L-Pop (3M ESPE, St. Paul, MN, USA): One-step self-etch	Water			
		Xeno III (Dentsply, Konstanz, Germany): Two-step self-etch	Ethanol, water			
Klein-Júnior [13]	2008	Adper Single Bond 2 (3M ESPE, St. Paul, MN, USA): Two-step etch-and-rinse	Ethanol, water	60 ± 2 °C	Human dentin (Permanent)	μTBS
		Prime & Bond 2.1 (Dentsply, Konstanz, Germany): Two-step etch-and-rinse	Acetone			
Marsiglio [21]	2012	Adper Scotchbond Multi-Purpose (3M ESPE, St. Paul, MN, USA): Three- step etch- and-rinse	Water	38 °C	Human dentin (Permanent)	μTBS
		Adper Single Bond 2 (3M ESPE, St. Paul, MN, USA): Two-step etch-and-rinse	Ethanol, water			
		Prime & Bond 2.1 (Dentsply, Mildford, Germany): Two-step etch-and-rinse	Acetone			
Moura [16]	2014	Adper SE Plus (3M ESPE; St. Paul, MN, USA): Two-step self-etch	Water	60 ± 2 °C	Human dentin (Permanent)	μTBS
		Clearfil 3S Bond (Kuraray Medical Inc, Tokyo, Japan): One-step self-etch	Water, ethanol			
		OptiBond All in-one (Kerr Co., Orange, CA, USA): One-step self-etch	Water, ethanol, and acetone			
		Silorane (3M ESPE; St. Paul, MN, USA): Two-step self-etch	Water, ethanol			
Ogura [20]	2012	Adper Easy Bond (3M ESPE, St. Paul, MN, USA): One-step self-etch	Ethanol, water	37 °C	Bovine dentin	Shear bond strength (SBS)
		Clearfil tri-S Bond (Kuraray Medical Inc., Tokyo, Japan): One-step self-etch	Ethanol, water			
		G-Bond Plus (GC Corp., Tokyo, Japan): One-step self-etch	Acetone, water			
Reis [12]	2010	Adper Single Bond 2 (3M ESPE, St. Paul, MN, USA): Two-step etch-and-rinse	Ethanol, water	60 ± 2 °C	Human dentin (Permanent)	μTBS
		Prime & Bond 2.1 (Dentsply, Mildford, Germany): Two-step etch-and-rinse	Acetone			
Riad [52]	2022	Adper Single Bond 2 (3M ESPE, St. Paul, MN, USA): Two-step etch-and-rinse	Ethanol, water	50 °C	Human dentin (Permanent)	SBS
		Single Bond Universal (3M ESPE, St. Paul, MN, USA): Universal adhesive applied in a self-etch mode	Ethanol, water			
Shiratsuchi [53]	2013	Bond Force (Tokuyama Dental, Tokyo, Japan): One-step self-etch	Isopropanol, water	37 °C	Bovine enamel	SBS
		Clearfil tri-S Bond (Kuraray Medical Inc., Tokyo, Japan): One-step self-etch	Ethanol, water			
		G-Bond Plus (GC Corp., Tokyo, Japan): One-step self-etch	Acetone, water			
Taguchi [14]	2018	Clearfil Bond SE ONE (Kuraray Noritake Dental Inc., Tokyo, Japan): One-step self-etch	Water, ethanol	80 ± 1 °C	Human dentin (Permanent)	μTBS
		Unifil Core EM Self-etch Bond (GC Corp., Tokyo, Japan): One-step self-etch	Water, ethanol			
		Estelink (Tokuyama Dental Corp., Tokyo, Japan): One-step self-etch	Water, ethanol, and acetone			
		Beauti Dual bond EX (Shofu Inc., Kyoto, Japan): One-step self-etch	Water, ethanol, and acetone			
Yonekura [55]	2020	Scotchbond Universal (3M ESPE, St. Paul, MN, USA): Universal adhesive used in a self-etch mode	Water, ethanol	60 ± 1 °C	Human dentin (Permanent)	μTBS
		Clearfil Bond SE ONE (Kuraray Noritake Dental Inc., Tokyo, Japan): One-step self-etch	Water, ethanol			
		Unifil Core EM Self-etch Bond (GC Corp. Tokyo, Japan): One-step self-etch	Water, acetone			
		Estelink (Tokuyama Dental Corp., Tokyo, Japan): One-step self-etch	Water, ethanol, and acetone			
Zimmer [56]	2022	Single Bond Universal (3M Oral Care, St. Paul, MN, USA): Universal adhesive system	Ethanol, water	50 °C	Human dentin (Permanent)	μTBS

**Table 3:** Demographic and study design data of the included studies regarding indirect substrates.

Study	Year	Material Tested and Category of the Material	Solvent Contained within the Material	Temperature of Warm Air Stream	Substrate Tested	Bond Strength Test
Baratto [39]	2015	Silano (Dentsply, Santiago, Chile) Silane (DMG, Hamburg, Germany): Silanes	Ethanol	50 ± 5 °C, 80 °C	Heat-pressed lithium disilicate glass-ceramic discs (IPS e.max Press; Ivoclar Vivadent AG, Schaan, Liechtenstein)	SBS
Carvalho [40]	2015	Clearfil Ceramic Primer (Kuraray Medical Inc., Tokyo, Japan): Silane	Ethanol	50 ± 5 °C	Ceramic (Vacumat, Vita Zahnfabrik)	μTBS
Colares [43]	2013	RelyX Ceramic Primer (3M ESPE, St. Paul, MN, USA): Silane	Ethyl alcohol, water	45 ± 5 °C	Crystallized lithium-disilicate-based glass blocks (IPSe.max CAD; Ivoclar Vivadent)	μTBS
Cotes [44]	2013	RelyX Ceramic Primer (3M ESPE; St. Paul, MN, USA): Silane	Ethyl alcohol, water	50 ± 5 °C	Ceramic block (VITA Zahnfabrik; Bad Säckingen, Germany)	μTBS
Fabianelli [46]	2010	Monobond-S (Ivoclar-Vivadent, Schaan, Liechtenstein): Pre-hydrolyzed silane coupling agent	Ethanol	100 °C	Leucite-reinforced ceramic blocks IPS Empress (Ivoclar Vivadent, Schaan, Liechtenstein)	μTBS
Kim [48]	2013	Porcelain Liner M (Sun Medical, Moriyama City, Japan): Two-component silane coupling agent	Ethanol	38 °C	Glass-fiber post (FRC Postec Plus (Ivoclar Vivadent AG, Schaan, Liechtenstein; and D.T. Light Post, BISCO Inc., Schaumburg, IL, USA)	SBS
Melo-Silva [49]	2012	Silane (3M ESPE, St. Paul, MN, USA)	Ethanol	70 °C	Ceramic-type (Y- TZP and feldspar)	SBS
Monticelli [50]	2006	Monobond-S (Ivoclar-Vivadent, Schaan, Liechtenstein): Pre-hydrolyzed silane coupling agent	Ethanol	38 °C	Quartz fiber posts (DT Light Post #2, RTD, St.Egéve, France)	μTBS
		Porcelain Liner M (Sun Medical Co., Ltd., Japan): Two-component silane coupling agent	Ethanol			
		Porcelain Silane (BJM Lab, Or-Yenuda, Israel): Pre-hydrolyzed silane coupling agent	Ethanol			
Novais [51]	2012	Silano (Angelus, Petrópolis, RJ, Brazil)	Ethanol	60 °C	Glass fiber posts (Exacto; Angelus, Londrina, PR, Brazil; size 2, 17.0 mm long × 1.50 mm diameter)	Push-out testing
		Prosil (FGM, Joinville, SC, Brazil)	Ethanol, water			
		RelyX Ceramic Primer (3M ESPE, St. Paul, MN, USA)	Ethyl alcohol, water			
		Silane coupling agent (Dentsply, Petrópolis, RJ, Brazil): Silanes	Ethanol, acetic acid			
Ramón-Leonardo [24]	2020	RelyX Ceramic Primer (3M ESPE, St. Paul, MN, USA)	Ethyl alcohol, water	100 °C	Lithium-disilicate-r-inforced glass ceramic (e.max CAD Ivoclar Vivadent, Schaan, Liechtenstein)	μSBS
		Monobond N (Ivoclar Vivadent, Schaan, Liechtenstein): Silanes	Ethanol			
de Rosatto [45]	2014	Silano (Angelus, Petrópolis, RJ, Brazil)	Ethanol	60 °C	Fiberglass posts (Exacto, Angelus)	Push-out testing
		Prosil (FGM, Joinville, SC, Brazil)	Ethanol, water			
		RelyX Ceramic Primer (3M ESPE, St. Paul, MN, USA)	Ethyl alcohol, water			
		Silane coupling agent (Dentsply, Petrópolis, RJ, Brazil): Silanes	Ethanol, acetic acid			
Shen [29]	2004	Monobond-S (Ivoclar-Vivadent, Schaan, Liechtenstein): Pre-hydrolyzed silane coupling agent	Ethanol	45 ±5 °C	Eris (Ivoclar Vivadent, Schaan, Liechtenstein) IPS Empress (Ivoclar Vivadent)	μTBS
Silva [54]	2013	Monobond-S (Ivoclar Vivadent AG, Schaan, Linchtenstein): Pre-hydrolyzed silane coupling agent	Ethanol	100 °C	Monocrystalline alumina premolar brackets (Pure^®^, OrthoTechnology, Tampa, FL, USA)	SBS
Yanakiev [23]	2017	Monobond Plus (Ivoclar Vivadent, Schaan, Lichtenstein): Silane coupling agent	Ethanol	38 °C, 50 °C, 100 °C, 120 °C	EX-3 veneering ceramic (Kuraray Noritake Dental, Japan)	Tensile bond strength (TBS)

**Table 4:** Methodological quality assessment.

Study	Specimen Randomization	Single Operator	Operator Blinded	Control Group	Standardized Specimens	Failure Mode	Manufacturer’s Instructions	Sample Size Calculation	Coefficient of Variation	Risk of Bias
Al-Salamony [38]	Yes	Yes	No	Yes	Yes	No	Yes	No	Yes	Medium
Baratto [39]	No	No	No	Yes	Yes	Yes	Yes	No	Yes	Medium
Carvalho [40]	Yes	No	No	Yes	Yes	Yes	Yes	Yes	Yes	Low
Carvalho [41]	Yes	No	No	Yes	Yes	Yes	Yes	No	Yes	Medium
Chen [42]	Yes	No	No	Yes	Yes	Yes	Yes	No	Yes	Medium
Colares [43]	Yes	No	No	Yes	No	Yes	Yes	No	Yes	Medium
Cotes [44]	Yes	No	No	Yes	Yes	Yes	Yes	No	Yes	Medium
Fabianelli [46]	Yes	No	No	Yes	Yes	Yes	Yes	No	Yes	Medium
Garcia [47]	Yes	No	No	Yes	Yes	No	Yes	No	Yes	Medium
Klein-Júnior [13]	Yes	Yes	No	Yes	Yes	Yes	Yes	No	Yes	Low
Kim [48]	No	No	No	Yes	Yes	No	Yes	No	Yes	Medium
Marsiglio [21]	Yes	No	No	Yes	Yes	Yes	Yes	No	Yes	Medium
Melo-Silva [49]	No	No	No	Yes	Yes	Yes	Yes	Yes	Yes	Medium
Monticelli [50]	No	No	No	Yes	Yes	Yes	Yes	Yes	Yes	Medium
Moura [16]	No	Yes	No	Yes	Yes	Yes	Yes	No	Yes	Medium
Novais [51]	Yes	No	No	Yes	Yes	Yes	Yes	No	Yes	Medium
Ogura [20]	No	No	No	Yes	Yes	Yes	Yes	No	Yes	Medium
Ramón-Leonardo [24]	Yes	Yes	No	Yes	Yes	Yes	Yes	No	Yes	Low
Reis [12]	No	Yes	No	Yes	Yes	Yes	Yes	No	Yes	Medium
Riad [52]	Yes	No	No	Yes	Yes	Yes	Yes	Yes	Yes	Low
de Rosatto [45]	No	No	No	Yes	Yes	Yes	Yes	No	Yes	Medium
Shen [29]	No	No	No	Yes	Yes	Yes	Yes	No	Yes	Medium
Shiratsuchi [53]	Yes	No	No	Yes	No	Yes	Yes	No	Yes	Medium
Silva [54]	Yes	Yes	No	Yes	Yes	No	Yes	No	Yes	Medium
Taguchi [14]	No	No	No	Yes	Yes	Yes	Yes	No	Yes	Medium
Yanakiev [23]	No	No	No	Yes	Yes	No	Yes	No	Yes	Medium
Yonekura [55]	No	No	No	Yes	Yes	Yes	Yes	No	Yes	Medium
Zimmer [56]	No	No	No	Yes	Yes	Yes	Yes	No	Yes	Medium

## Data Availability

The data presented in this study are available on reasonable request from the authors (R.B. and L.H.).
